# 3,5-Di­bromo-4-carbamoyl­benzoic acid 2-propanol monosolvate

**DOI:** 10.1107/S2414314621003916

**Published:** 2021-04-20

**Authors:** Wayland E. Noland, Ryan J. Herzig, Renee J. Fox, Kenneth J. Tritch

**Affiliations:** a Department of Chemistry, University of Minnesota, 207 Pleasant St SE, Minneapolis, MN 55455, USA; University of Aberdeen, Scotland

**Keywords:** crystal structure, hydrogen bond, di­bromo­arene, solvate, benzamide

## Abstract

In the title solvated crystal, C_8_H_5_Br_2_NO_3_·C_3_H_8_O, the acid mol­ecules form inversion dimers by pairwise N—H⋯O hydrogen bonds between carbamoyl groups and the carboxyl and carbamoyl groups link to form head-to-tail inversion dimers. The 2-propanol hydroxyl group inter­poses between adjacent head-tail pairs, resulting in *C*_3_^3^(10) chains of hydrogen bonds propagating along [100]. The mol­ecules of 2-propanol are disordered over two sets of sites in a 0.598 (8):0.402 (8) ratio. The best-fit planes of the carbamoyl group and benzene ring are inclined by 88.26 (11)°. This is a greater inclination than was previously reported with CH_3_, Cl, F or H in place of the Br atoms, although those analogues did not have a *para* carboxyl group.

## Abstract 2

Mol­ecules of the title compound are linked by hydrogen-bond dimerization of the amido group, and a chain of hydrogen bonds between 2-propanol and the carboxyl and amido groups that propagates parallel to [100].

## Graphical abstract

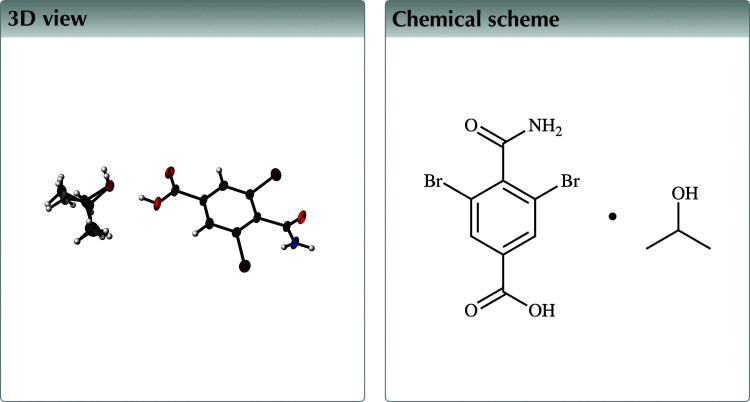

## Structure description

**Figure 1:**
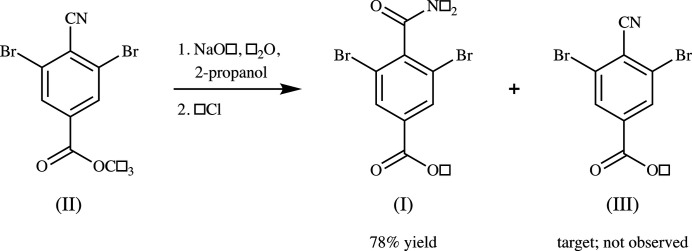
The synthesis of (I).

Although the structure of 2-bromo­benzamide is reported twice (Izumi & Okamoto, 1972 ▸; Gulyás *et al.*, 2015 ▸) in the current version of the Cambridge Structural Database (version 5.42, Nov 2020; Groom *et al.*, 2016 ▸), no 2,6-di­bromo- or 4-carboxyl­benzamides were found. The title compound (I) is an example of both classes, and was accidentally prepared in an attempt to selectively hydrolyze the ester group of a cyano ester (II) (Fig. 1 ▸). The target was cyano acid (III), for a study in our laboratory involving co-crystals of (III) with anthracene (Noland *et al.*, 2017 ▸).

**Figure 2:**
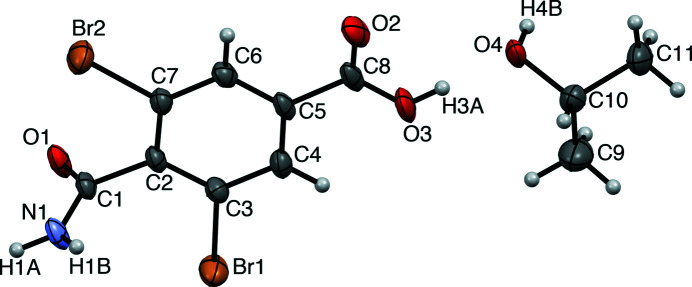
The mol­ecular structure of (I), with atomic numbering and displacement ellipsoids at the 50% probability level. For clarity, only the major disorder component of 2-propanol is shown.

**Figure 3:**
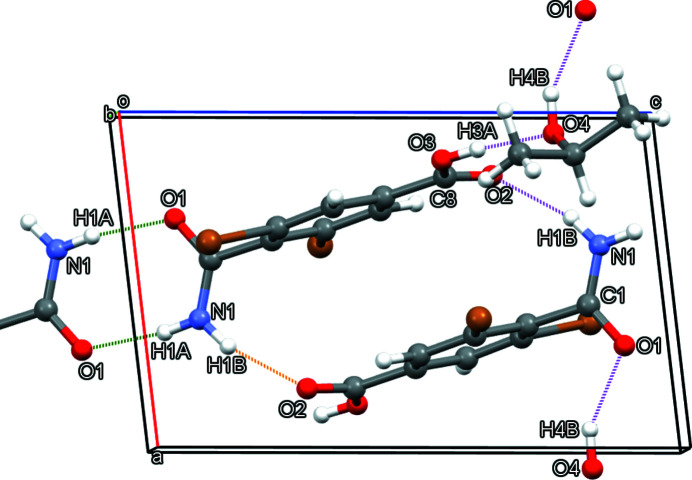
Hydrogen bonding in the crystal of (I), viewed along [010]. For clarity, the minor component and the lower-left mol­ecule of 2-propanol are omitted from the unit cell. The dashed green lines represent amide dimerization. The dashed magenta lines represent the hydrogen bonds that form chains along [100]. The dashed orange line and its magenta counterpart (O2⋯N1) illustrate head-to-tail dimerization.

In the title crystal (Fig. 2 ▸), mol­ecules of (I) form typical amide inversion dimers based on pairwise N1—H1*A*⋯O1 hydrogen bonds (Table 1 ▸; Fig. 3 ▸). The carboxyl groups do not homo-dimerize. Instead, they participate in amido-carb­oxy N1—H1*B*⋯O2 hydrogen bonding that forms head-to-tail inversion dimers about the center of the unit cell. The solvent mol­ecule of 2-propanol inter­poses between H3*A* of the acid group and O1 of the amide group, forming an O3—H3*A*⋯O4—H4*B*⋯O1 hydrogen-bonded chain. Excluding amide dimerization, these hydrogen bonds collectively form 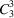 (10) chains propagating along [100]. The 2-propanol mol­ecule is disordered over two sets of sites in a 0.598 (8):0.402 (8) ratio. The Br atoms and benzene ring do not participate in any short inter­actions.

**Figure 4:**
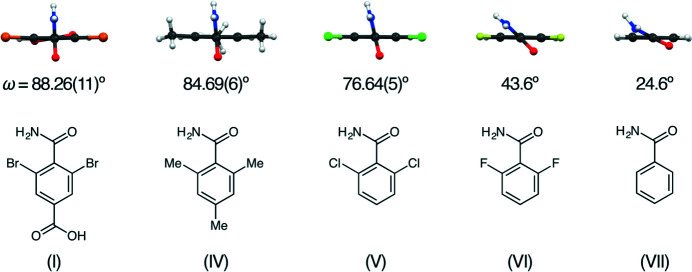
Substituted benzamides, in their crystals, viewed along carbamoyl group *ipso* bonds. The listed dihedral angles (*ω*) are between the best-fit planes of the respective benzene rings and carbamoyl groups.

A dihedral angle of 88.26 (11)° is observed between the best-fit planes of the carbamoyl group (O1/N1/C1) and the benzene ring (C2–C7; Fig. 4 ▸). The corresponding angle is also shown for 2,4,6-trimethyl- (IV; Gdaniec *et al.*, 2004 ▸), 2,6-di­chloro- (V; Mukherjee *et al.*, 2013 ▸), 2,6-di­fluoro- (VI; Rauf *et al.*, 2006 ▸), and unsubstituted (VII; Blake *et al.*, 1972 ▸) benzamides. Although none of these reported crystals contain a *para*-carboxyl group, crystal (I) fits the expected trend. The *ortho* Br atoms cause a carbamoyl inclination slightly larger than the inclination observed with *ortho* methyl groups, which is in turn larger than inclinations caused by *ortho* Cl, F, or H atoms.

## Synthesis and crystallization

**3,5-Di­bromo-4-carbamoyl­benzoic acid (I)**: a portion of compound (II) (589 mg, Fig. 1 ▸) taken from our prior study (Noland *et al.*, 2017 ▸) was placed in a round-bottomed flask with water (5 ml), 2-propanol (5 ml) and NaOH (186 mg). The resulting mixture was refluxed for 1 h, and then cooled to 290 K. Hydro­chloric acid (6 *M*) was added dropwise until the pH of the mixture was less than 4. An off-white precipitate was collected by suction filtration, and then triturated with 2-propanol, giving a white powder (476 mg, 78%), m.p. 528–529 K. ^1^H NMR (500 MHz, DMSO-*d*_6_) *δ* 13.705 (*s*, 1H, H3*A*), 8.124 (*s*, 1H, H1*A* or H1*B*), 8.069 (*s*, 2H, H4*A*, H6*A*), 7.887 (*s*, 1H, H1*B* or H1*A*); ^13^C NMR (126 MHz, DMSO-*d*_6_) *δ* 166.8 (1 C, C1 or C8), 164.5 (1 C, C8 or C1), 144.3 (1 C, C5), 133.4 (1 C, C2), 132.0 (2 C, C4, C6), 119.7 (2 C, C3, C7); IR (KBr, cm^−1^) 3442, 3314, 3185, 3088, 2921, 2487, 1719, 1648, 1604, 1542, 1371, 1270, 901, 743; MS (ESI, *m*/*z*) [*M* − H]^−^ calculated for C_8_H_5_^81^Br^79^BrNO_3_ 321.8543, found 321.8549.

**Crystallization:** a portion of the white powder was dissolved in refluxing 2-propanol and the resulting mixture was incrementally cooled to 268 K over 6 h. Crystals were collected by deca­ntation, and then washed with 2-propanol.

## Refinement

Crystal data, data collection and structure refinement details are summarized in Table 2 ▸.

## Supplementary Material

Crystal structure: contains datablock(s) I. DOI: 10.1107/S2414314621003916/hb4381sup1.cif

Structure factors: contains datablock(s) I. DOI: 10.1107/S2414314621003916/hb4381Isup2.hkl

Supporting information file. DOI: 10.1107/S2414314621003916/hb4381Isup3.cml

CCDC reference: 1525812

Additional supporting information:  crystallographic information; 3D view; checkCIF report

## Figures and Tables

**Table 1:** Hydrogen-bond geometry (Å, °)

*D*—H⋯*A*	*D*—H	H⋯*A*	*D*⋯*A*	*D*—H⋯*A*
O3—H3*A*⋯O4	0.84 (1)	1.74 (1)	2.580 (12)	174 (3)
O3—H3*A*⋯O4′	0.84 (1)	1.72 (2)	2.560 (19)	173 (3)
N1—H1*A*⋯O1^i^	0.88 (1)	2.06 (1)	2.929 (2)	171 (3)
N1—H1*B*⋯O2^ii^	0.88 (1)	2.08 (1)	2.931 (3)	164 (3)
O4—H4*B*⋯O1^iii^	0.84 (1)	2.05 (3)	2.802 (11)	149 (5)
O4′—H4*C*⋯O1^iii^	0.84 (1)	1.90 (5)	2.606 (18)	141 (7)

Symmetry codes: (i) 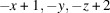 ; (ii) 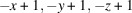 ; (iii) 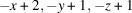 .

**Table 2:** Experimental details

Crystal data	
Chemical formula	C_8_H_5_Br_2_NO_3_·C_3_H_8_O
*M*_r_	383.04
Crystal system, space group	Triclinic, *P* 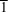
Temperature (K)	173
*a*, *b*, *c* (Å)	7.2866 (5), 8.5212 (6), 12.1832 (8)
α, β, γ (°)	71.376 (1), 81.745 (1), 83.682 (1)
*V* (Å^3^)	707.76 (8)
*Z*	2
Radiation type	Mo *K*α
μ (mm^−1^)	5.73
Crystal size (mm)	0.40 × 0.38 × 0.16

Data collection	
Diffractometer	Bruker APEXII CCD
Absorption correction	Multi-scan (*SADABS*; Krause *et al.*, 2015 ▸)
*T*_min_, *T*_max_	0.486, 0.746
No. of measured, independent and observed [*I* > 2σ(*I*)] reflections	8139, 3128, 2683
*R*_int_	0.021
(sin θ/λ)_max_ (Å^−1^)	0.645

Refinement	
*R*[*F* ^2^ > 2σ(*F* ^2^)], *wR*(*F* ^2^), *S*	0.026, 0.065, 1.04
No. of reflections	3128
No. of parameters	201
No. of restraints	151
H-atom treatment	H atoms treated by a mixture of independent and constrained refinement
Δρ_max_, Δρ_min_ (e Å^−3^)	0.63, −0.90

Computer programs: *APEX3* and *SAINT* (Bruker, 2018 ▸), *SHELXT2014* (Sheldrick, 2015*a* ▸), *SHELXL2018/3* (Sheldrick, 2015*b* ▸), *Mercury* (Macrae *et al.*, 2020 ▸) and *publCIF* (Westrip, 2010 ▸).

## full crystallographic data

### Crystal data

**Table:**

C_8_H_5_Br_2_NO_3_·C_3_H_8_O	*F*(000) = 376
*M**_r_* = 383.04	*D*_x_ = 1.797 Mg m^−^^3^
Triclinic, *P*1	Melting point: 528 K
*a* = 7.2866 (5) Å	Mo *K*α radiation, λ = 0.71073 Å
*b* = 8.5212 (6) Å	Cell parameters from 2971 reflections
*c* = 12.1832 (8) Å	θ = 2.6–27.3°
α = 71.376 (1)°	µ = 5.73 mm^−^^1^
β = 81.745 (1)°	*T* = 173 K
γ = 83.682 (1)°	Block, colourless
*V* = 707.76 (8) Å^3^	0.40 × 0.38 × 0.16 mm
*Z* = 2	

### Data collection

**Table:**

Bruker APEXII CCD diffractometer	2683 reflections with *I* > 2σ(*I*)
Radiation source: sealed tube	*R*_int_ = 0.021
φ and ω scans	θ_max_ = 27.3°, θ_min_ = 1.8°
Absorption correction: multi-scan (SADABS; Krause *et al.*, 2015)	*h* = −9→9
*T*_min_ = 0.486, *T*_max_ = 0.746	*k* = −11→10
8139 measured reflections	*l* = −15→15
3128 independent reflections	

### Refinement

**Table:**

Refinement on *F*^2^	151 restraints
Least-squares matrix: full	Hydrogen site location: mixed
*R*[*F*^2^ > 2σ(*F*^2^)] = 0.026	H atoms treated by a mixture of independent and constrained refinement
*wR*(*F*^2^) = 0.065	*w* = 1/[σ^2^(*F*_o_^2^) + (0.0296*P*)^2^ + 0.5063*P*] where *P* = (*F*_o_^2^ + 2*F*_c_^2^)/3
*S* = 1.04	(Δ/σ)_max_ = 0.001
3128 reflections	Δρ_max_ = 0.63 e Å^−^^3^
201 parameters	Δρ_min_ = −0.90 e Å^−^^3^

### Special details

**Table:**

Geometry. All esds (except the esd in the dihedral angle between two l.s. planes) are estimated using the full covariance matrix. The cell esds are taken into account individually in the estimation of esds in distances, angles and torsion angles; correlations between esds in cell parameters are only used when they are defined by crystal symmetry. An approximate (isotropic) treatment of cell esds is used for estimating esds involving l.s. planes. Angle of C2/C3/C4/C5/C6/C7 vs. N1/C1/O1 below Least-squares planes (x,y,z in crystal coordinates) and deviations from them (* indicates atom used to define plane) 6.9373 (0.0020) x - 1.2809 (0.0074) y + 2.6193 (0.0107) z = 5.8890 (0.0086) * 0.0044 (0.0015) C2 * -0.0085 (0.0016) C3 * 0.0048 (0.0015) C4 * 0.0030 (0.0015) C5 * -0.0070 (0.0015) C6 * 0.0033 (0.0015) C7 Rms deviation of fitted atoms = 0.0055 1.3629 (0.0122) x + 7.5482 (0.0167) y + 8.7867 (0.0367) z = 9.5168 (0.0262) Angle to previous plane (with approximate esd) = 88.256 ( 0.113 ) * 0.0000 (0.0000) N1 * 0.0000 (0.0000) C1 * 0.0000 (0.0000) O1 Rms deviation of fitted atoms = 0.0000
Refinement. A direct-methods solution was calculated, followed by full-matrix least squares / difference Fourier cycles. All H atoms were placed in calculated positions (C—H = 0.95–1.00 Å, N—H = 0.88 Å, O—H = 0.84 Å) and refined as riding atoms with *U*_iso_(H) set to 1.2 or 1.5*U*_eq_(C/N/O).

### Fractional atomic coordinates and isotropic or equivalent isotropic displacement parameters (Å^2^)

**Table:**

	*x*	*y*	*z*	*U*_iso_*/*U*_eq_	Occ. (<1)
Br1	0.61784 (4)	0.53550 (3)	0.84990 (2)	0.04348 (9)	
Br2	0.61751 (4)	0.03664 (3)	0.63196 (2)	0.04169 (9)	
O1	0.6947 (2)	0.0875 (2)	0.90019 (14)	0.0332 (4)	
O2	0.8164 (3)	0.6055 (2)	0.30554 (14)	0.0405 (4)	
O3	0.8603 (3)	0.7832 (2)	0.39910 (15)	0.0416 (4)	
H3A	0.891 (4)	0.846 (3)	0.3317 (11)	0.050*	
N1	0.3942 (3)	0.1688 (3)	0.87692 (17)	0.0327 (4)	
H1A	0.355 (4)	0.097 (3)	0.9439 (12)	0.039*	
H1B	0.312 (3)	0.227 (3)	0.8307 (19)	0.039*	
C1	0.5730 (3)	0.1757 (3)	0.84327 (18)	0.0252 (4)	
C2	0.6307 (3)	0.3004 (3)	0.72637 (17)	0.0241 (4)	
C3	0.6625 (3)	0.4624 (3)	0.71657 (19)	0.0265 (4)	
C4	0.7250 (3)	0.5743 (3)	0.61079 (19)	0.0278 (5)	
H4A	0.748438	0.683825	0.606128	0.033*	
C5	0.7527 (3)	0.5239 (3)	0.51214 (18)	0.0264 (5)	
C6	0.7196 (3)	0.3647 (3)	0.51800 (19)	0.0276 (5)	
H6A	0.737311	0.331352	0.449673	0.033*	
C7	0.6603 (3)	0.2545 (3)	0.62524 (19)	0.0256 (4)	
C8	0.8134 (3)	0.6414 (3)	0.3944 (2)	0.0308 (5)	
O4	0.9365 (13)	0.9719 (9)	0.1879 (11)	0.0313 (15)	0.598 (8)
H4B	1.0530 (14)	0.964 (8)	0.184 (5)	0.047*	0.598 (8)
C9	0.872 (3)	1.205 (2)	0.2603 (12)	0.060 (2)	0.598 (8)
H9A	0.820755	1.320374	0.241438	0.091*	0.598 (8)
H9B	1.000209	1.198652	0.278449	0.091*	0.598 (8)
H9C	0.796644	1.135893	0.327854	0.091*	0.598 (8)
C10	0.8716 (7)	1.1441 (5)	0.1583 (4)	0.0345 (13)	0.598 (8)
H10A	0.739971	1.152928	0.141246	0.041*	0.598 (8)
C11	0.9845 (12)	1.2460 (14)	0.0493 (7)	0.0429 (18)	0.598 (8)
H11A	0.979250	1.201108	−0.014763	0.064*	0.598 (8)
H11B	1.113936	1.241251	0.064208	0.064*	0.598 (8)
H11C	0.933135	1.361577	0.028151	0.064*	0.598 (8)
O4'	0.978 (2)	0.9791 (14)	0.1997 (18)	0.0313 (15)	0.402 (8)
H4C	1.049 (8)	0.954 (10)	0.146 (5)	0.047*	0.402 (8)
C9'	0.883 (5)	1.202 (4)	0.2791 (18)	0.060 (2)	0.402 (8)
H9D	0.887410	1.321374	0.265058	0.091*	0.402 (8)
H9E	0.938938	1.140568	0.350651	0.091*	0.402 (8)
H9F	0.753869	1.174455	0.287647	0.091*	0.402 (8)
C10'	0.9900 (11)	1.1550 (7)	0.1784 (5)	0.0304 (17)	0.402 (8)
H10B	1.123331	1.176385	0.174482	0.036*	0.402 (8)
C11'	0.9228 (19)	1.250 (2)	0.0639 (10)	0.0429 (18)	0.402 (8)
H11D	0.793361	1.227577	0.065230	0.064*	0.402 (8)
H11E	1.000083	1.215842	0.001592	0.064*	0.402 (8)
H11F	0.931011	1.369041	0.049729	0.064*	0.402 (8)

### Atomic displacement parameters (Å^2^)

**Table:**

	*U*^11^	*U*^22^	*U*^33^	*U*^12^	*U*^13^	*U*^23^
Br1	0.0693 (2)	0.03621 (15)	0.02540 (13)	−0.00969 (12)	0.00169 (12)	−0.01126 (10)
Br2	0.05996 (18)	0.02835 (13)	0.03767 (15)	−0.01118 (11)	−0.00300 (12)	−0.00992 (11)
O1	0.0278 (8)	0.0364 (9)	0.0226 (8)	−0.0049 (7)	−0.0010 (6)	0.0086 (7)
O2	0.0473 (10)	0.0395 (10)	0.0201 (8)	0.0044 (8)	0.0024 (7)	0.0058 (7)
O3	0.0468 (10)	0.0323 (9)	0.0294 (9)	−0.0094 (8)	0.0080 (8)	0.0101 (7)
N1	0.0295 (10)	0.0359 (11)	0.0200 (10)	−0.0038 (8)	0.0008 (8)	0.0080 (8)
C1	0.0314 (11)	0.0240 (10)	0.0155 (10)	−0.0062 (9)	0.0000 (8)	0.0007 (8)
C2	0.0260 (10)	0.0237 (10)	0.0170 (10)	−0.0044 (8)	−0.0003 (8)	0.0016 (8)
C3	0.0309 (11)	0.0270 (11)	0.0186 (10)	−0.0023 (9)	−0.0007 (8)	−0.0039 (8)
C4	0.0284 (11)	0.0238 (10)	0.0259 (11)	−0.0044 (9)	−0.0024 (9)	0.0005 (9)
C5	0.0221 (10)	0.0276 (11)	0.0195 (10)	−0.0012 (8)	0.0004 (8)	0.0053 (8)
C6	0.0295 (11)	0.0299 (11)	0.0184 (10)	0.0003 (9)	−0.0010 (8)	−0.0022 (9)
C7	0.0281 (11)	0.0231 (10)	0.0218 (10)	−0.0033 (8)	−0.0011 (8)	−0.0019 (8)
C8	0.0222 (10)	0.0311 (12)	0.0246 (11)	0.0025 (9)	0.0016 (9)	0.0080 (9)
O4	0.026 (4)	0.0233 (10)	0.034 (3)	−0.0051 (17)	0.008 (3)	0.0027 (10)
C9	0.091 (4)	0.0453 (19)	0.042 (4)	−0.0020 (19)	0.009 (4)	−0.016 (3)
C10	0.036 (3)	0.027 (2)	0.035 (2)	0.0005 (17)	−0.0004 (19)	−0.0050 (17)
C11	0.059 (6)	0.0292 (15)	0.032 (2)	−0.001 (4)	0.000 (3)	0.0010 (18)
O4'	0.026 (4)	0.0233 (10)	0.034 (3)	−0.0051 (17)	0.008 (3)	0.0027 (10)
C9'	0.091 (4)	0.0453 (19)	0.042 (4)	−0.0020 (19)	0.009 (4)	−0.016 (3)
C10'	0.039 (4)	0.020 (3)	0.030 (3)	−0.007 (2)	−0.009 (2)	−0.001 (2)
C11'	0.059 (6)	0.0292 (15)	0.032 (2)	−0.001 (4)	0.000 (3)	0.0010 (18)

### Geometric parameters (Å, º)

**Table:**

Br1—C3	1.893 (2)	C9—C10	1.492 (10)
Br2—C7	1.890 (2)	C9—H9A	0.9800
O1—C1	1.236 (3)	C9—H9B	0.9800
O2—C8	1.213 (3)	C9—H9C	0.9800
O3—C8	1.311 (3)	C10—C11	1.517 (8)
O3—H3A	0.839 (3)	C10—H10A	1.0000
N1—C1	1.311 (3)	C11—H11A	0.9800
N1—H1A	0.880 (3)	C11—H11B	0.9800
N1—H1B	0.880 (3)	C11—H11C	0.9800
C1—C2	1.515 (3)	O4'—C10'	1.448 (11)
C2—C3	1.389 (3)	O4'—H4C	0.840 (3)
C2—C7	1.390 (3)	C9'—C10'	1.497 (13)
C3—C4	1.385 (3)	C9'—H9D	0.9800
C4—C5	1.382 (3)	C9'—H9E	0.9800
C4—H4A	0.9500	C9'—H9F	0.9800
C5—C6	1.381 (3)	C10'—C11'	1.492 (12)
C5—C8	1.501 (3)	C10'—H10B	1.0000
C6—C7	1.385 (3)	C11'—H11D	0.9800
C6—H6A	0.9500	C11'—H11E	0.9800
O4—C10	1.437 (8)	C11'—H11F	0.9800
O4—H4B	0.840 (3)		
			
C8—O3—H3A	110 (2)	H9A—C9—H9C	109.5
C1—N1—H1A	119.3 (18)	H9B—C9—H9C	109.5
C1—N1—H1B	121.2 (18)	O4—C10—C9	109.7 (8)
H1A—N1—H1B	119 (2)	O4—C10—C11	110.9 (7)
O1—C1—N1	124.32 (19)	C9—C10—C11	112.8 (10)
O1—C1—C2	118.97 (19)	O4—C10—H10A	107.8
N1—C1—C2	116.71 (19)	C9—C10—H10A	107.8
C3—C2—C7	117.58 (19)	C11—C10—H10A	107.8
C3—C2—C1	121.60 (19)	C10—C11—H11A	109.5
C7—C2—C1	120.79 (19)	C10—C11—H11B	109.5
C4—C3—C2	121.8 (2)	H11A—C11—H11B	109.5
C4—C3—Br1	118.38 (17)	C10—C11—H11C	109.5
C2—C3—Br1	119.85 (16)	H11A—C11—H11C	109.5
C5—C4—C3	118.9 (2)	H11B—C11—H11C	109.5
C5—C4—H4A	120.5	C10'—O4'—H4C	106 (6)
C3—C4—H4A	120.5	C10'—C9'—H9D	109.5
C6—C5—C4	121.02 (19)	C10'—C9'—H9E	109.5
C6—C5—C8	117.7 (2)	H9D—C9'—H9E	109.5
C4—C5—C8	121.3 (2)	C10'—C9'—H9F	109.5
C5—C6—C7	118.9 (2)	H9D—C9'—H9F	109.5
C5—C6—H6A	120.6	H9E—C9'—H9F	109.5
C7—C6—H6A	120.6	O4'—C10'—C9'	108.5 (13)
C6—C7—C2	121.8 (2)	O4'—C10'—C11'	109.7 (10)
C6—C7—Br2	118.33 (17)	C9'—C10'—C11'	113.5 (14)
C2—C7—Br2	119.82 (15)	O4'—C10'—H10B	108.3
O2—C8—O3	124.9 (2)	C9'—C10'—H10B	108.3
O2—C8—C5	122.0 (2)	C11'—C10'—H10B	108.3
O3—C8—C5	113.2 (2)	C10'—C11'—H11D	109.5
C10—O4—H4B	109 (5)	C10'—C11'—H11E	109.5
C10—C9—H9A	109.5	H11D—C11'—H11E	109.5
C10—C9—H9B	109.5	C10'—C11'—H11F	109.5
H9A—C9—H9B	109.5	H11D—C11'—H11F	109.5
C10—C9—H9C	109.5	H11E—C11'—H11F	109.5
			
O1—C1—C2—C3	−91.0 (3)	C4—C5—C6—C7	−0.9 (3)
N1—C1—C2—C3	89.3 (3)	C8—C5—C6—C7	−178.9 (2)
O1—C1—C2—C7	86.7 (3)	C5—C6—C7—C2	0.9 (3)
N1—C1—C2—C7	−93.0 (3)	C5—C6—C7—Br2	−179.53 (16)
C7—C2—C3—C4	−1.3 (3)	C3—C2—C7—C6	0.1 (3)
C1—C2—C3—C4	176.4 (2)	C1—C2—C7—C6	−177.6 (2)
C7—C2—C3—Br1	178.09 (16)	C3—C2—C7—Br2	−179.40 (16)
C1—C2—C3—Br1	−4.2 (3)	C1—C2—C7—Br2	2.8 (3)
C2—C3—C4—C5	1.3 (3)	C6—C5—C8—O2	7.2 (3)
Br1—C3—C4—C5	−178.05 (16)	C4—C5—C8—O2	−170.8 (2)
C3—C4—C5—C6	−0.2 (3)	C6—C5—C8—O3	−173.2 (2)
C3—C4—C5—C8	177.7 (2)	C4—C5—C8—O3	8.9 (3)

### Hydrogen-bond geometry (Å, º)

**Table:**

*D*—H···*A*	*D*—H	H···*A*	*D*···*A*	*D*—H···*A*
O3—H3*A*···O4	0.84 (1)	1.74 (1)	2.580 (12)	174 (3)
O3—H3*A*···O4′	0.84 (1)	1.72 (2)	2.560 (19)	173 (3)
N1—H1*A*···O1^i^	0.88 (1)	2.06 (1)	2.929 (2)	171 (3)
N1—H1*B*···O2^ii^	0.88 (1)	2.08 (1)	2.931 (3)	164 (3)
O4—H4*B*···O1^iii^	0.84 (1)	2.05 (3)	2.802 (11)	149 (5)
O4′—H4*C*···O1^iii^	0.84 (1)	1.90 (5)	2.606 (18)	141 (7)

Symmetry codes: (i) −*x*+1, −*y*, −*z*+2; (ii) −*x*+1, −*y*+1, −*z*+1; (iii) −*x*+2, −*y*+1, −*z*+1.
